# Distinctive Metabolomics Patterns Associated With Insulin Resistance and Type 2 Diabetes Mellitus

**DOI:** 10.3389/fmolb.2020.609806

**Published:** 2020-12-14

**Authors:** Xinyun Gu, Mohammed Al Dubayee, Awad Alshahrani, Afshan Masood, Hicham Benabdelkamel, Mahmoud Zahra, Liang Li, Anas M. Abdel Rahman, Ahmad Aljada

**Affiliations:** ^1^Department of Chemistry, University of Alberta, Edmonton, AB, Canada; ^2^Department of Medicine, College of Medicine, King Saud bin Abdulaziz University for Health Sciences, King Abdullah International Medical Research Center, Ministry of National Guard Health Affairs, Riyadh, Saudi Arabia; ^3^Obesity Research Center, College of Medicine, King Saud University, Riyadh, Saudi Arabia; ^4^Department of Biochemistry and Molecular Medicine, College of Medicine, Alfaisal University, Riyadh, Saudi Arabia; ^5^Department of Genetics, King Faisal Specialist Hospital and Research Center, Riyadh, Saudi Arabia; ^6^Department of Chemistry, Memorial University of Newfoundland, St. John’s, NL, Canada

**Keywords:** type 2 diabetes mellitus, insulin resistance, obesity, untargeted metabolomics profiling, clinical metabolic panel, chemical isotope labeling liquid chromatography

## Abstract

Obesity is associated with an increased risk of insulin resistance (IR) and type 2 diabetes mellitus (T2DM) which is a multi-factorial disease associated with a dysregulated metabolism and can be prevented in pre-diabetic individuals with impaired glucose tolerance. A metabolomic approach emphasizing metabolic pathways is critical to our understanding of this heterogeneous disease. This study aimed to characterize the serum metabolomic fingerprint and multi-metabolite signatures associated with IR and T2DM. Here, we have used untargeted high-performance chemical isotope labeling (CIL) liquid chromatography-mass spectrometry (LC-MS) to identify candidate biomarkers of IR and T2DM in sera from 30 adults of normal weight, 26 obese adults, and 16 adults newly diagnosed with T2DM. Among the 3633 peak pairs detected, 62% were either identified or matched. A group of 78 metabolites were up-regulated and 111 metabolites were down-regulated comparing obese to lean group while 459 metabolites were up-regulated and 166 metabolites were down-regulated comparing T2DM to obese groups. Several metabolites were identified as IR potential biomarkers, including amino acids (Asn, Gln, and His), methionine (Met) sulfoxide, 2-methyl-3-hydroxy-5-formylpyridine-4-carboxylate, serotonin, L-2-amino-3-oxobutanoic acid, and 4,6-dihydroxyquinoline. T2DM was associated with dysregulation of 42 metabolites, including amino acids, amino acids metabolites, and dipeptides. In conclusion, these pilot data have identified IR and T2DM metabolomics panels as potential novel biomarkers of IR and identified metabolites associated with T2DM, with possible diagnostic and therapeutic applications. Further studies to confirm these associations in prospective cohorts are warranted.

## Introduction

Obesity is considered the most crucial factor in the development of several metabolic diseases such as T2DM. The prevalence of obesity in the Middle East is increasing, where about 70% of males above 20 years old are overweight compared to females (74%) in Saudi Arabia (Ng et al., 2014). Obesity and T2DM are conditions that are interlinked biochemically, metabolically, and at multiple levels making it difficult to discern the differences in their pathologies. Although closely interconnected, not all individuals with obesity develop diabetes and remain metabolically healthy, while a majority of patients diagnosed with T2DM are obese (predominant central obesity). The strong bidirectional relationship existing between obesity and T2DM is causally linked by IR (Al-Goblan et al., 2014) as a result of an increased chronic low-grade inflammation and oxidative stress, which are characteristics of both states. IR is characterized by a decreased tissue responsiveness to circulating insulin levels leading to defects in uptake and oxidation of glucose, a decrease in glycogen synthesis, decreased ability to suppress lipid oxidation, and the existence of a pro-oxidant state. The presence of IR far precedes the onset and presentation of the clinical symptoms of T2DM due to HG, delaying its prediction, diagnosis, and management by several years (Sas et al., 2015).

Insulin resistance, commonly observed in patients with obesity, affects multiple organs, including the adipose tissue, muscle, and liver, and attenuates insulin signaling pathways. In obese individuals, adipose tissue releases bigger amounts of NEFAs that promote triglyceride accumulation, resulting in worsening IR and β-cell dysfunction (Scheen, 2003). Shortly after an acute increase in plasma NEFA levels in humans, IR starts to develop. On the other hand, when the level of plasma NEFA decreases, as in antilipolytic agent used cases, peripheral insulin uptake improves. It has also been proposed the connection of NEFA and fatty acids delivery and intracellular metabolism to the levels of intracellular content of fatty acid metabolites such as diacylglycerol (DAG), which activates a serine (Ser)/threonine kinase cascade leading to Ser/threonine phosphorylation of IRS-1 and INSR substrate-2 (IRS-2), and a reduced ability of these molecules to activate PI3K (Snel et al., 2012). Subsequently, events downstream of INSR signaling are diminished due to the lipotoxicity giving rise to IR in obese individuals. Initiation of IR forms the first phase in the pathogenesis of T2DM followed sequentially by elevations in plasma glucose levels (that stimulate β-cells to secrete higher amounts of insulin), oxidative stress (that accelerates β-cell insufficiency) exacerbating the existing HG, ultimately leading to apoptosis (β-cell death) and development of overt T2DM (Cernea and Dobreanu, 2013).

Metabolomics has recently become a powerful method to measure subtle biochemical changes in several diseases (Adamski, 2016). Several metabolites associated with IR and obesity have been identified in T2DM including BCAAs, AAA, mannose, fructose, α-hydroxybutyrate, and phospholipids (Wang et al., 2011; Ferrannini et al., 2013; Yu et al., 2016). The onset of T2DM is relatively long, and symptoms of T2DM can occur at a very late stage without acute metabolic disturbances, making it difficult for early diagnosis (Sas et al., 2015). The current clinical practice for T2DM diagnoses, such as fasting glucose and glucose tolerance tests, lacks efficient early diagnose of T2DM. Identification of sensitive *in vivo* biomarkers that could reflect the early onset of T2DM would be crucial for the identification of high-risk asymptomatic diabetic individuals for better prevention. To identify metabolomics patterns for IR and T2DM individuals, a high-performance CIL LC-MS was utilized in this study. Our goal is to identify potential metabolic biomarkers for IR and T2DM, other than HOMA-IR and HG. CIL is used to modify the chemical and physical properties of metabolites for much-improved separation and enhanced detection sensitivity, thereby increasing the number of detectable metabolites (Jacob et al., 2019a; Dahabiyeh et al., 2020). Using differential isotope labeling also provides more accurate and precise quantification of metabolite concentration differences in comparative samples (i.e., relative quantification) (Jacob et al., 2019a; Dahabiyeh et al., 2020).

## Materials and Methods

### Subjects

All subjects were recruited from a primary healthcare hospital located at King Abdulaziz Medical City in Riyadh, Saudi Arabia. All subjects underwent a medical check-up at the Department of Medicine and were screened for medical history. The anthropometric measurements included weight, height, waist, and hip circumferences. The study participants comprised of three groups: 30 adults of normal weight, 26 obese adults, and 16 adults newly diagnosed with T2DM. Exclusion criteria included (1) patients with coronary event or procedure (myocardial infarction, unstable angina, coronary artery bypass surgery, or coronary angioplasty) in the previous 3 months; (2) patient on steroids; (3) hepatic disease (transaminase > 3 times normal); (4) renal impairment (serum creatinine > 1.5 mg/dL); (5) history of drug or alcohol abuse; (6) participation in any other concurrent clinical trials; (7) any other life-threatening diseases; and (8) use of an investigational agent within 30 days of study. Institutional review board (IRB) approval was obtained from both King Abdulaziz Medical City Ethics Committee (Protocol # RC12/105), and King Faisal Specialist Hospital and Research Center (KFSHRC) (RAC# 2170 013), and all study participants signed a written informed consent form. All volunteers were properly instructed to fast for 12 h before the day appointed for vein puncture.

### Chemicals and Reagents

Liquid chromatography-mass spectrometry grade reagents, including water, ACN, methanol, and FA, were purchased from Fisher Scientific (Ottawa, ON). ^13^C-DnsCl was available from Nova Medical Testing, Inc. (Edmonton, Canada) with the procedures published previously (Zhao et al., 2019).

### Metabolomic Profiling Workflow

Supplementary Figure S1 shows the schematic of the overall metabolomics analysis workflow. Each sample was derivatized by ^12^C-DnsCl, while a pooled sample generated by mixing of aliquots of all individual samples was labeled by ^13^C-DnsCl. The ^13^C-labeled pool sample served as an internal standard for all ^12^C-labeled individual samples. The sample amount of each sample was normalized using the LC-UV method (Wu and Li, 2012). The ^12^C-labeled individual sample was mixed with the same mole amount of ^13^C-labeled pool. The mixture was injected onto LC-MS. All the labeled metabolites were detected as peak pairs on mass spectra. The peak area ratios were used for quantitative metabolomics analysis; the same ^13^C-labeled pool was spiked into all ^12^C-labeled individual samples, and thus the peak ratio values of a labeled metabolite in different samples reflected the concentration differences of this metabolite in these samples. In other words, every ^12^C-labeled metabolite from an individual sample had its corresponding ^13^C-labeled metabolite in the pooled sample as a reference, resulting in high accuracy for relative quantification (Jacob et al., 2019a; Dahabiyeh et al., 2020).

### Serum Samples and Dansylation Labeling

Serum samples including lean control (*n* = 30), obese (*n* = 26), and T2DM (*n* = 16) were collected and stored at −80°C. A 15 μL sample was used and the metabolites were extracted by protein precipitation with 45 μL of methanol. After 2 h incubation at −20°C, 45 μL of supernatant was dried and then mixed with 25 μL of water, 12.5 μL of ACN, 12.5 μL of sodium carbonate/sodium bicarbonate buffer, and 25 μL of ^12^C-DnsCl or ^13^C-DnsCl (18 mg/mL in ACN). The mixture was incubated at 40°C for 45 min and 5 μL of 250 mM NaOH were added and incubated for 10 min at 40°C. Twenty-five μL of 425 mM FA in 1:1 ACN/H_2_O was added to consume excess NaOH.

### LC-UV

Before LC-MS injections, sample normalization was performed to minimize variations in the total sample amount of individual samples when comparing samples. A step-gradient LC-UV method measured the total concentration of dansyl labeled metabolites (Wu and Li, 2012). In brief, 5 μL of the labeled sample was injected into a Phenomena’s Kinetes C18 column (2.1 mm × 5 cm, 1.7 μm particle size, 100 Å pore size) connected to a Waters ACQUITY UPLC system (Waters, Milford, MA, United States). Mobile phase A was 0.1% (v/v) FA in 5% (v/v) ACN, and mobile phase B was 0.1% (v/v) FA in ACN. The 6.5 min LC gradient including: *t* = 0 min, 0% B; *t* = 1 min, 0% B; *t* = 1.1 min, 95% B; *t* = 2.6 min, 95% B; *t* = 3.1 min, 0% B, and the flow rate was 0.45 mL/min. PDA detector was operated at 338 nm. The area under the peak representing the total concentration of dansyl-labeled metabolites was integrated using Waters Empower (V6.00).

### LC-MS

Each sample was labeled by ^12^C-DnsCl and mixed in equal mole amount with a ^13^C-labeled pool sample based on the quantification results from LC-UV analysis. The samples were analyzed by a Dionex Ultimate 3000 UHPLC System (Thermo Scientific, Sunnyvale, CA, United States) connected to Maxis II quadrupole time-of-flight (Q-TOF) mass spectrometer (Bruker, Billerica, MA, United States). The analytes were separated using a reversed-phase Eclipse Plus C18 column (2.1 mm × 10 cm, 1.8 μm particle size, 95 Å pore size) (Agilent Inc., Santa Clara, CA, United States). Mobile phase A was 0.1% (v/v) FA in 5% (v/v) ACN, and solvent B was 0.1% (v/v) FA in ACN. The LC gradient was: *t* = 0 min, 20% B; *t* = 3.5 min, 35% B; *t* = 18 min, 65% B; *t* = 21 min, 99% B; *t* = 34 min, 99% B, and flow rate of 0.18 mL/min. MS conditions were as follows: polarity, positive; dry temperature, 230°C; dry gas, 8 L/min; capillary voltage, 4500 V; nebulizer, 1.0 bar; endplate offset, 500 V; spectra rate, 1.0 Hz.

The quality control (QC) sample was prepared by mixing the ^12^C- and ^13^C-labeled pooled samples in equal mole. A QC injection was performed every 15 LC-MS sample runs. In total, there were 14 QC samples injected and analyzed. Peak pairs with ratio values having >±25% RSD in the QC samples were filtered out.

### Data Analysis

The MS spectra of the detected analytes were converted into.cvs files using Bruker Daltonics Data Analysis 4.3 software. The raw data generated from multiple LC-MS runs were processed by peak picking, peak pairing, and peak-pair filtering to remove redundant peaks (IsoMS; Zhou et al., 2014). IsoMS files from each injection were aligned together based on the peak’s accurate mass and retention time to generate the aligned file. The missing peak pair information in aligned files was re-extracted from raw data by Zerofill software (Huan and Li, 2015). The final metabolite-intensity data file was used for statistical analysis after normalization and/or scaling. The PLS-DA was performed by MetaboAnalyst^1^.

Metabolite identification was carried out using the three-tier metabolite identification approach (Zhao et al., 2019). In tier 1, peak pairs were searched against a labeled metabolite library (CIL Library) based on accurate mass and retention time. The CIL Library (i.e., dansyl amines and phenols) contains 711 experimental entries, including metabolites and dipeptides (Huan et al., 2015). In tier 2, linked identity library (LI Library) was used for identification of the remaining peak pairs. LI Library includes metabolic-pathway-related metabolites (more than 7000 entries extracted from the KEGG database), providing high-confidence putative identification results based on accurate mass and predicted retention time matches. In tier 3, the remaining peak pairs were searched, based on accurate mass match, against the MyCompoundID (MCID) library composed of 8021 known human endogenous metabolites (zero-reaction library) and their predicted metabolic products from one metabolic reaction (375,809 compounds) (one-reaction library) and two metabolic reactions (10,583,901 compounds) (two-reaction library) (Li et al., 2013). The identified features were processed for building the IR and T2DM models using Multiple Professional Profiler (MPP) Software (Agilent Inc., Santa Clara, CA, United States) to construct the Venn diagrams, clustering heat maps, and the profiles.

## Results

### Demographic Data of Study Participants

Demographic data of study participants are summarized in Table 1. The study groups are lean, obese, and newly diagnosed obese with T2DM. Both obese and T2DM groups were significantly older than the normal weight group. Few subjects from groups 2 and 3 were on statins or other cholesterol-lowering agents, angiotensin-converting enzyme inhibitors (ACE-I) or other anti-hypertensives, non-steroidal anti-inflammatory drugs, or anti-oxidants and they were on stable doses for the last 2 months of their participation in the study. T2DM group had significantly higher LDL and Trig and HDL when compared to the healthy disease-free groups.

**TABLE 1:** Demographic data of study participants.

	**Lean (*n* = 30)**	**Obese (*n*** = **26)**	**T2DM (*n* = 16)**
Age (years)	25.7 ± 5.77	36.2 ± 12.12†	49.4 ± 12.12†‡
Geniler (FM)	(11/19)	(17/9)	(4/12)
BMI (kg/m^2^)	23.0 ± 1.48	38.8+8.59†	32.7 ± 7.61†
Glucose (mmol/L)	5.1 ± 0.5	5.4 ± 0.67	10.2 ± 4.99†‡
HbA1c	–	5.3 ± 1.73	8.7 ± 2.82†
LDL(mmol/L)	2.5 ± 0.94	3.0 ± 0.81	3.6 ± 0.72†
HDL (mmol/L)	1.35 ± 0.16	1.18 ± 0.27†	1.01 ± 0.21†
Trig (mmol/L)	0.83 ± 0.33	1.26 ± 0.61	2.01 ± 1.04†
Insulin (μU/mL)	4.59 ± 2.04	9.98 ± 6.17†	7.44 ± 8.53
HOMA-IR	1.03 ± 0.56	2.30 ± 1.44†	2.80 ± 2.55

*Results are presented as Mean ± SD. †*p* < 0.05 vs normal weight subjects. ‡*P* < 0.05 vs obese.*

### Metabolomics Results

Based on the unique characteristics of the peak pair of the CIL LC-MS method, 3633 peak pairs were detected in the participants’ samples (the full data can be found in https://www.ebi.ac.uk/metabolights/MTBLS2098) (Supplementary Table S1). The IsoMS software filtered out redundant peak pairs such as those from adduct ions, dimers, multimers, etc., to retain only one peak pair ([M + H]^+^) for each metabolite. Thus, the number of peak pairs detected reflects the number of detected metabolites. From the detected peak pairs, 216 metabolites were positively identified using both retention time and accurate mass searching against the labeled metabolite library (CIL Library). One hundred thirty-five peak pairs were putatively identified based on accurate mass and predicted retention time matches by searching against the LI Library. Six hundred and eleven and 1296 metabolites were putatively matched with the zero-reaction and one-reaction library via accurate mass only by searching against the MCID library, respectively. Thus, 62.2% of the 3633 peak pairs detected were either identified or matched, which shows the significant coverage of the submetabolome using the dansylation labeling LC-MS method for the serum samples analyzed in this study.

### Statistical Analysis Between Study Groups

Multivariate statistical analysis was performed to analyze the serum metabolome dataset. PLS-DA was first performed to reveal the distinct separation between the groups visually. The metabolome dataset was analyzed to see the separation between lean and obese groups, as shown in Supplementary Figure S2A, where the clusters of two groups were separated with *Q*^2^ = 0.737 and *R*^2^ = 0.972. Univariate analysis to further analyze the metabolome changes using volcano plots was performed on the metabolome set. In the volcano plot, the *x*-axis is the fold change (FC) of the obese group over the lean control group, and the *y*-axis is the *p*-value from the *t*-test for comparing the two groups., The *q*-value (false discovery rate) less than 0.05, and FC > 1.5 (or FC < 0.67) were used to determine metabolites with significance, that has been calculated using R Script. Herein, the cutoff *p*-value equals *q*-value, which is 0.05. The FC criterion chosen was based on the technical accuracy and reproducibility, i.e., for dansylation LC-MS, the errors and RSD values are less than ± 25%. Thus, we conservatively used ± 50% change as the criterion. In Supplementary Figure S2B, a total of 189 metabolites were dysregulated. Among them, 78 metabolites were up-regulated (FC > 1.5) and 111 metabolites were down-regulated (FC < 0.67) comparing obese to lean group. By searching against our dansyl standard library using these 189 metabolites, 30 of them were positively identified.

A clear separation was observed with *Q*^2^ = 0.885 and *R*^2^ = 0.985 from the PLS-DA score plot of T2DM and obese groups in Supplementary Figure S2C. The clear separation illustrates that obese and T2DM groups experienced some significant metabolome alterations. From the volcano plot showed in Supplementary Figure S2D, 459 metabolites were up-regulated (FC > 1.5), and 166 metabolites were down-regulated (FC < 0.67) comparing T2DM to obese groups. The cut-off *p*-value here is 0.038 (when *q*-value = 0.05). Sixty-seven metabolites out of 625 were positively identified using the Dnsyl library. PLS-DA analysis was also performed to the lean vs T2DM. Two clusters were well separated on the PLS-DA score plot with *Q*^2^ = 0.809 and *R*^2^ = 0.977 as shown in Supplementary Figure S2E. From the volcano plot showed in Supplementary Figure S2F, 189 metabolites were up-regulated (FC > 1.5), and 117 metabolites were down-regulated (FC < 0.67) comparing lean to T2DM groups. The cut-off *p*-value here is 0.068 (when *q*-value = 0.05). Three hundred and five metabolites were common between the three groups and were used in the downstream analysis for building the IR and T2DM models.

### Metabolic Profile for Study Confounder

**FIGURE 1:**
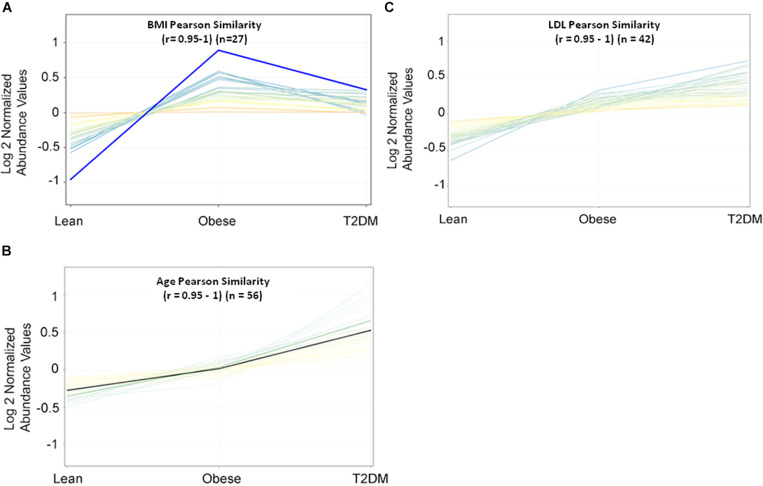
Pearson similarity tests (*r* = 0.95–1) were filtered out with 27, 56, and 42 metabolites as **(A)** BMI-dependent; **(B)** age-dependent, and **(C)** LDL-C-dependent, respectively.

Metabolomics expression in human serum is highly sensitive to specific physiological changes such as age, BMI, LDL-cholesterol (LDL-C), etc. (Jacob et al., 2019b). In this study, BMI, age, and LDL-C are the main confounders that were considered in the downstream data analysis. The values of these confounders for the study participants were integrated into the metabolomics dataset. Pearson similarity test (*R* = 0.95–1) reveals 27 metabolites depend on BMI (Figure 1A), and 56 metabolites on age (Figure 1B), while 42 depend on LDL-C (Figure 1C). These confounders-related metabolites were excluded from IR and T2DM metabolic profiles. HOMA-IR and glucose-dependent metabolites were also determined using the same similarity approach as the other diabetic confounders and used to compare them with the final metabolic pattern of IR and T2DM.

### IR Metabolic Pattern

**FIGURE 2:**
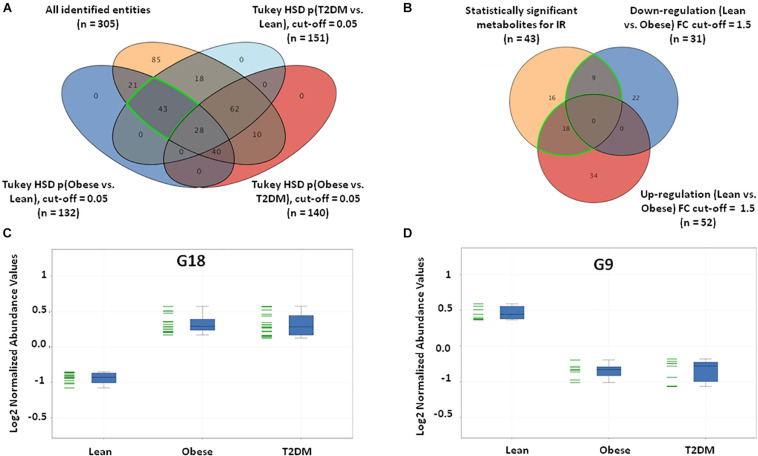
IR metabolic model based on the positively identified features. **(A)** A Venn diagram showing that out of 3633 detected features, only 305 (^~^8%) metabolites were positively identified. 43 (G43) significant metabolites were considered a metabolic pattern for IR, where they were statistically significant between lean vs obese and lean vs T2D, and insignificant between obese vs T2D (FDR-corrected *p*-value < 0.05). **(B)** A Venn diagram showing the number of the up- and down-regulated metabolites after fold change analysis (cutoff l.5) on the significant metabolites (G43) (nine and 18 metabolites, respectively). **(C)** The expression profile of G18 and **(D)** G9 metabolites.

Insulin resistance metabolic pattern was built using a model where dysregulated metabolites in obesity remained unchanged in T2DM compared to the lean group. After one-way ANOVA and Tukey honest significant difference (HSD) analysis, significantly different metabolites for each pair of groups were demonstrated in a Venn diagram by applying IR metabolic model on overall detected features, 351 identified and unidentified metabolites fell within this IR pattern (Supplementary Figure S3A), only 66 feature were up-regulated and 100 down-regulated in both obese and T2DM compared to lean as shown in Supplementary Figures S3B–D. The identified metabolites between the study groups (*n* = 305) were further analyzed to extract the IR metabolic pattern (Figure 2A). IR metabolic group were 43 metabolites that are statistically significant between both the lean vs obese and lean vs T2DM groups, and insignificant between obese vs T2DM. Figure 2B shows the breakdown of the 43 metabolites based on FC analysis (FC > 1.5 or <0.67), where 18 metabolites (G18) were up-regulated in both obese and T2DM compared to lean group (Figure 2C), while nine metabolites (G9) were down-regulated in both obese and T2DM compared to lean (Figure 2D). Among the identified IR metabolic panel, the up- and down-regulated metabolites (G18, and G9, respectively) were further analyzed to exclude BMI-, age, and LDL-C-related metabolites (Supplementary Figures S4A,B). Only nine metabolites were down-regulated in obesity and T2DM and are independent of these three confounders (Supplementary Figure S4B).

### T2DM Metabolomics Pattern

**FIGURE 3:**
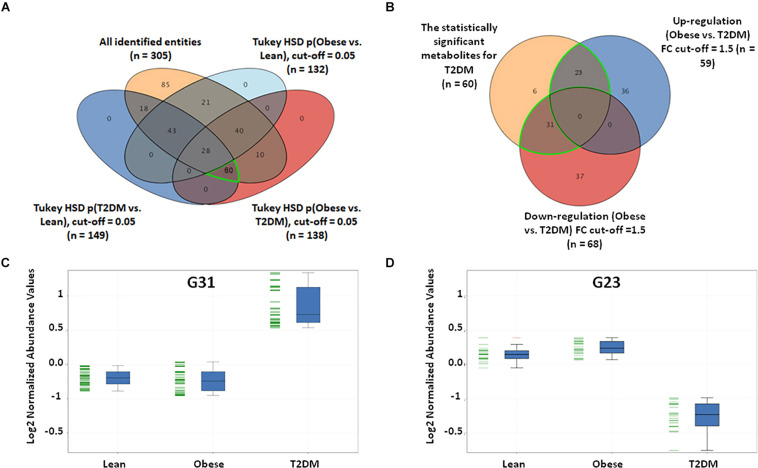
T2DM metabolic model based on the identified features only. **(A)** Among the positively identified metabolites, 62 features were considered T2DM metabolic profile, where they are statistically significant between lean and T2DM, and obese and T2DM, and insignificant between lean and obese (FDR-corrected *p*-value < 0.051). **(B)** Fold change analysis (cutoff 13) separates these 62 significant metabolites into up- and down-regulated in T2DM compared to both lean and obese groups (31 and 23 metabolites, respectively). **(C)** Representative profile of the up-regulated (G31) and **(D)** down-regulated (G23) metabolites.

**FIGURE 4:**
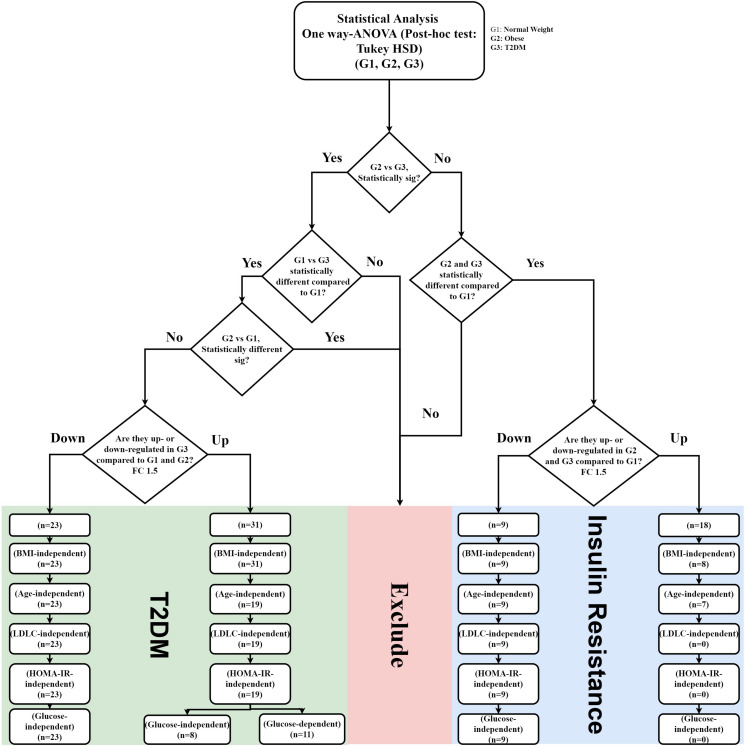
IR and T2DM metabolic panel data mining scheme. IR metabolic model was generated based on the fold change difference (FC cutoff 1.5) and statistically significant features between lean vs obese and lean vs T2D, and insignificance between obese vs T2D (FDR-corrected *p*-value < 0.05; *n* = 27 metabolites; 18 up-regulated, and nine down-regulated) T2DM metabolic panels were built based on fold change (FC cutoff 1.5), and statistical significance between lean and T2DM, and obese and T2DM, and insignificance between lean and obese (FDR-corrected *p*-value < 0.05) (*n* = 54 metabolites: 31 were up-regulated, and 23 down-regulated). These panels underwent several filtration stages to exclude BMI, age, and LDL-C effects. Eventually, the remaining metabolites in each panel were correlated with HOMA-IR and glucose levels.

In this study, a metabolomics pattern for T2DM has been determined by extracting the metabolites that significantly unchanged between the lean and obese groups and significantly dysregulated in T2DM compared to both lean and obese groups. As shown in Supplementary Figure S5A, out of 3633 detected features, 605 metabolites were dysregulated in T2DM compared to both lean and obese groups based on one-way ANOVA Tukey HSD cutoff (FDRp < 0.05). A total number of 529 significantly changed metabolites were filtered out based on FC analysis (FC > 1.5 or < 0.67) (Supplementary Figure S5B). One hundred eighty metabolites were down-regulated (G180) (Supplementary Figure S5C), and 349 (G349) were up-regulated (Supplementary Figure S5D) in T2DM compared to both lean and obese groups. Applying the same analysis on the identified molecules (*n* = 305), 62 metabolites were dysregulated in T2DM compared to both lean and obese groups (FDRp < 0.05), as shown in Figure 3A. Fifty-six metabolites out of 60 were significantly dysregulated based on FC analysis (FC > 1.5 or < 0.67) (Figure 3B), where 31 metabolites were up-regulated (Figure 3C), and 23 down-regulated (Figure 3D) in T2DM compared to other groups. After applying the confounder filters (BMI, age, and LDL-C) on these 54 metabolites (Supplementary Figures S6A,B), T2DM metabolic profile, 19, and 23 metabolites were up-regulated and down-regulated as BMI, age, and LDL-C-independent metabolites, respectively, as summarized in Figure 4.

### Biomarkers Evaluation for IR and T2DM Metabolic Patterns

**FIGURE 5:**
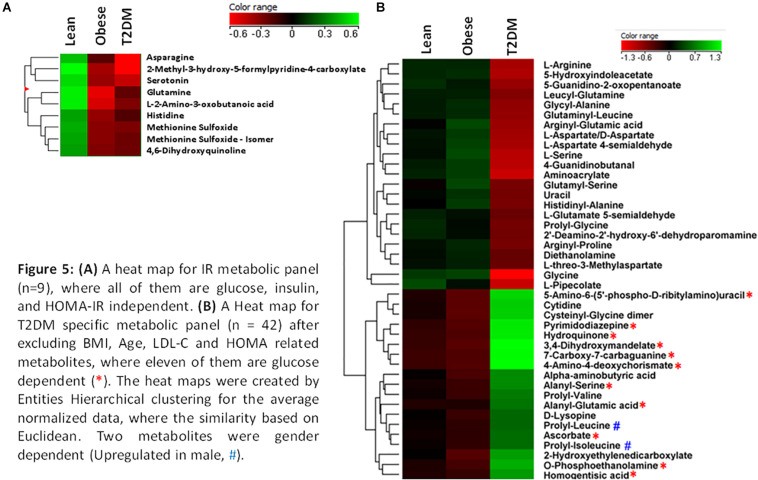
**(A)** A heat map for IR metabolic panel (*n* = 9), where all of them are glucose, insulin, and HOMA-IR independent. **(B)** A heat map for T2DM specific metabolic panel (*n* = 42) after excluding BMI, age, LDL-C, and HOMA related metabolites, where 11 of them are glucose dependent (*). The heat maps were created by entities hierarchical clustering for the average normalized data, where the similarity based on Euclidean. Two metabolites were gender dependent (up-regulated in male, #).

**FIGURE 6:**
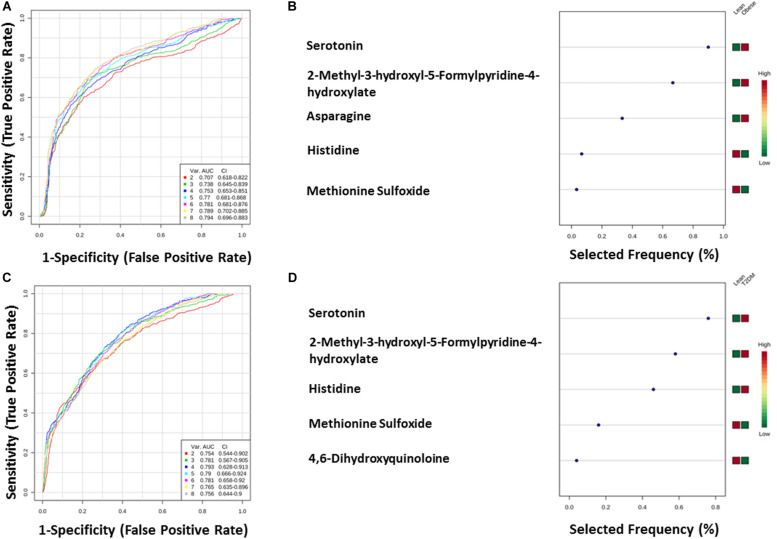
**(A,B)** Lean vs obese ROC analysis for IR top five metabolites. **(C,D)** Lean vs T2DM.

The nine metabolites of IR metabolic pattern are independent of glucose, HOMA-IR, and insulin as shown in Supplementary Figures S7A–C. On the other hand, the 42 metabolites of T2DM metabolic pattern were found to be HOMA-IR- and insulin-independent. However, only 11 metabolites were found to be glucose-dependent and up-regulated in T2DM compared to other groups (Supplementary Figure S7A). Heat maps that show cluster analysis of the entire average expression of each metabolite for IR specific panels (*n* = 9) (Figure 5A) and T2DM specific (*n* = 42) (Figure 5B) were generated after excluding all confounder related metabolites. The glucose-dependent metabolites are highlighted in these heat maps, which were created by Entities Hierarchical clustering for the average normalized data, and the similarity-based on Pearson. Also, two metabolites were gender-dependent and are up-regulated in males (prolyl-leucine and prolyl-isoleucine). The nine metabolites that represent IR metabolic pattern were analyzed as potential biomarkers, where the area under the curve (AUC) of the receiver operating characteristic (ROC) analysis was found 0.77 for the top changed five metabolites; serotonin, 2-methyl-3-hydroxy-5-formylpyridine-4-carboxylate, Asn, His, and methionine (Met) sulfoxide, when the comparison was done between the lean and obese groups (Figures 6A,B). Another comparison for the ability of this panel to predict IR in T2DM patients was performed for the same set of metabolites and found five metabolites (serotonin, 2-methyl-3-hydroxy-5-formylpyridine-4-carboxylate, His, Met sulfoxide, and 4, 6-dihydroxyquinoline) to have AUC 0.79 (Figures 6C,D).

**FIGURE 7:**
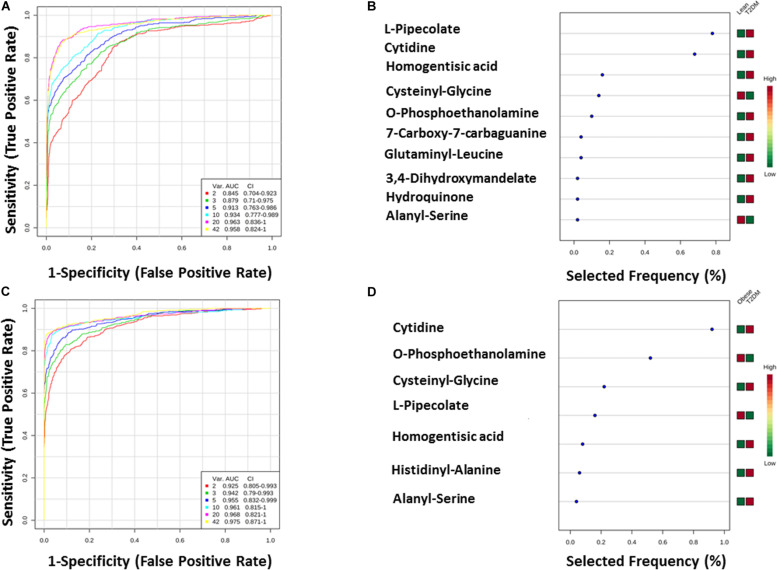
**(A,B)** Lean vs T2DM ROC analysis for T2DM top 10 metabolites. **(C,D)** Obese vs T2DM.

The T2DM metabolic panel with 42 metabolites was evaluated for being used as potential biomarkers using the AUC of the ROC analysis. Pipecolate, cytidine, homogentistic acid, cystenyl-glycine, phosphoethanolamin, 7-caraboxy-7-carbaguanidine, glutaminyl-leucine, 3,4-dihydroxymandelate, hydroquinone, and alanyl-Ser were found to be the highest to predict hyperglycemic diabetic patients from lean and obese with AUC 0.958 and 0.975, respectively.

**FIGURE 8:**
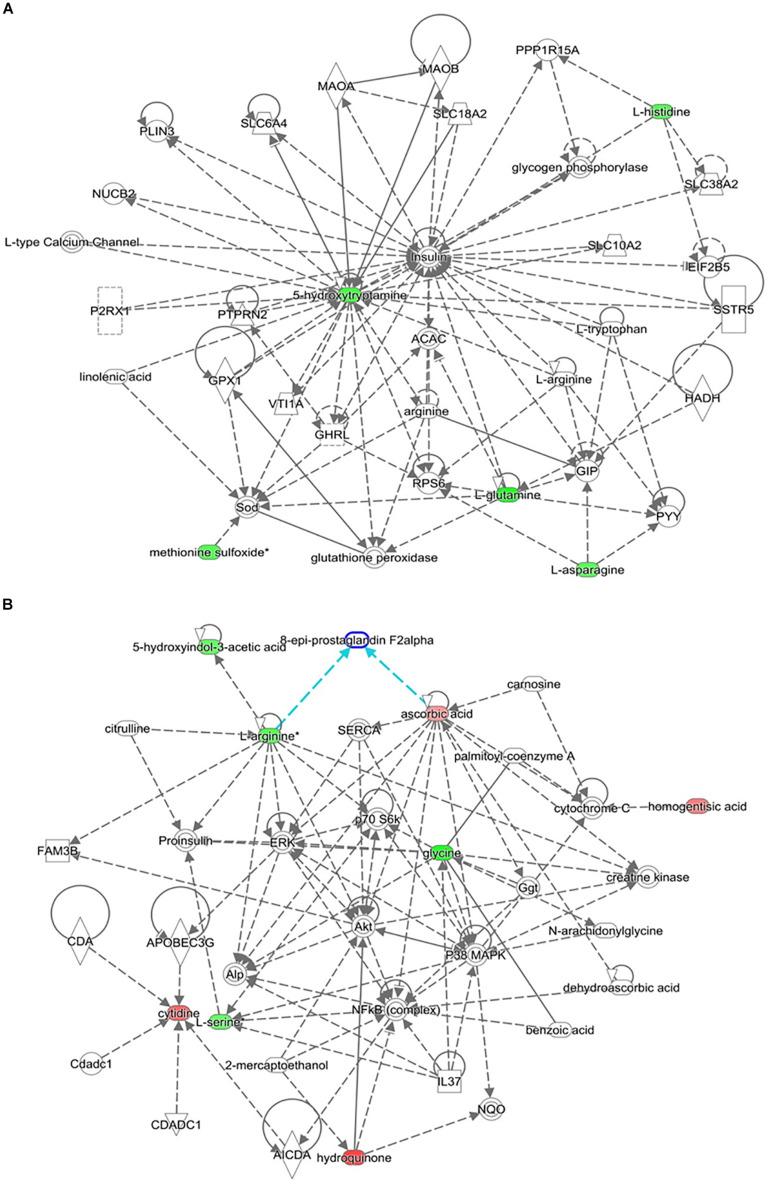
Depicted top scoring I PA metabolomic networks. **(A)** “Cell-to-cell signaling and interaction, molecular transport, small molecule biochemistry “for IR metabolomic panel and **(B)** “cellular compromise, lip id metabolism, small molecule biochemistry” for T2DM metabolomic panel. The dotted lines indicate indirect and the straight lines indicate direct relationships. Nodes colored red represent up-regulation and green represent down-regulation. The interaction networks were generated through the use of IPA (QIAGEN Inc., https://www.qiagenbioinformatics.com/products/ingenuity-pathway-analysis/).

The top-scoring IPA metabolomic networks “cell-to-cell signaling and interaction, molecular transport, small molecule biochemistry” (Figure 8A), and “cellular compromise, lipid metabolism, small molecule biochemistry” (Figure 8B), are depicted for IR and T2DM metabolic patterns, respectively.

## Discussion

Insulin resistance is observed when higher than normal insulin concentrations are needed to achieve normal metabolic responses or when normal insulin concentrations fail to achieve a normal metabolic response (Kahn, 1978; Campbell et al., 1988). IR can be identified earlier than insulin secretion failure and is not always associated with the development of diabetes when islet cell secretion can keep up with normal insulin demand. Many methods and indices are available for the estimation of IR. At present, the most reliable reference methods available for estimating IR are hyperinsulinemic-euglycemic clamp and intravenous glucose tolerance test. The glucose clamp approach has several limitations such as time-consuming, labor-intensive, expensive, and requires an experienced operator to manage the technical difficulties. Other simple methods, from which indices can be derived, include homeostasis model assessment (HOMA-IR), quantitative insulin sensitivity check index (QUICKI), and Matsuda index developed by Matsuda and DeFronzo (1999). These indices are used in epidemiological and clinical studies to predict diabetes development in a non-diabetic population. HOMA-IR is a model of the relationship between insulin and glucose dynamics that predicts fasting steady-state insulin and glucose concentrations for a wide range of possible combinations of IR and β-cell function. HOMA-IR values inversely connected to insulin sensitivity (Gutch et al., 2015). Nevertheless, HOMA-IR has limitations in subjects with a lower BMI, a lower β-cell function, and high fasting glucose levels such as lean T2DM with insulin secretory defects (Kang et al., 2005). Recently, Quantose IR Test has been introduced commercially. Quantose IR Test is a fasting blood test that measures a panel of biomarkers comprised of a small organic acid [alpha-hydroxybutyric acid (AHB), two lipids (oleic acid and linoleoylglycerophosphocholine (LGPC)], and insulin. In our present study, the top five metabolites; serotonin, 2-methyl-3-hydroxy-5-formylpyridine-4-carboxylate, Asn, His, and Met sulfoxide were found to have a significantly high discriminatory capacity in identifying IR. Aside from these, we also identified three amino acids (Gln, Asn, and His) and 4,6 dihydroxyquinoline [product of tryptophan (Trp) metabolism] to be associated with the IR metabolic profile. A high-fat diet in animal models reduced the levels of Gln and Asn (gluconeogenic amino acids) and 4,6-dihydroxyquinoline (Liu et al., 2017). Higher levels of Gln and His (suppressor of hepatic gluconeogenesis) are known to be significantly associated with a lower risk for incident T2DM (Kimura et al., 2013; Chen et al., 2019) while lower levels of circulating Asn are associated with increased BMI, IR, and HG (Banerji, 2015; Ottosson et al., 2018). Consistent with these findings, we found a decrease in the levels of Asn in obese T2DM compared to the obese and the lean groups, while the levels of Gln and His were lowered more in the obese than the obese T2DM patients.

Several studies have shown a correlation between certain amino acids and the development of diabetes years later. The mechanism by which elevations in plasma of certain amino acids links to the development of T2DM is currently unclear (Yamada et al., 2015; Chen et al., 2019; Vangipurapu et al., 2019). Glutamate (Glu) was the most strongly associated metabolite with T2DM, followed by increased levels of BCAA (Ottosson et al., 2018). Glu and Asn were both associated with a composite endpoint of developing T2DM or coronary artery disease (CAD; Ottosson et al., 2018). On the other hand, high Gln concentrations were associated with a decreased risk of incident T2DM (Chen et al., 2019) and decreased blood glucose in adolescents with T1DM after exercise while IR was unaltered during the euglycemic clamp (Torres-Santiago et al., 2017). Gln supplementation also reduced waist circumference in overweight and obese humans and improved insulin sensitivity in DIO Wistar rats (Abboud et al., 2019). Similarly, His oral supplementation improved IR (DiNicolantonio et al., 2018). His metabolism in T2DM may affect insulin sensitivity. His metabolism by the gut microbiota, in some T2DM patients, increases imidazole propionate levels which can decrease insulin sensitivity (Koh et al., 2018). Reduction of Gln, Asn, and His in obese and T2DM in this study is consistent with the previous reports of their role in increasing insulin sensitivity. Metabolites detected in the serum were decreased in obese and T2DM groups compared to lean subjects. Prominent decreases were also observed for metabolites from amino acids including 4,6-dihydroxyquinoline, Met sulfoxide, and L-2-amino-3-oxobutanoic acid. It is interesting to note that 4,6-dihydroxyquinoline was reported to be inhibited in high-fat diet-fed rats compared to normal diet controls (Liu et al., 2017). On the other hand, Met is one of the most susceptible to reactive oxygen species (ROS), resulting in both S and R diasteroisomeric forms (oxidation) of Met sulfoxide. Two Met residues in serum albumin (Met-111 and Met-147) are highly oxidized to Met sulfoxide in patients with diabetes (Suzuki et al., 2016) and the higher Met sulfoxide content in apoA-I from diabetic patients is consistent with lipid peroxidation products levels in plasma (Brock et al., 2008). Oxidative damage, mainly Met sulfoxide residues apolipoprotein B100 of LDL, was also increased in T2DM (Rabbani et al., 2010). The data on Met sulfoxide in this study contradict these studies and require further examination to elucidate the reduction of Met sulfoxide and its relation to IR.

Other serum metabolites that were reduced in IR include 2-methyl-3-hydroxy-5-formylpyridine-4-carboxylate, and serotonin or 5-hydroxytryptamine (5-HT). Reduction in 2-methyl-3-hydroxy-5-formylpyridine-4-carboxylate, an intermediate metabolite in vitamin B6 metabolism, observed in this study is consistent with the fact that vitamin B6 has been reported to help regulate blood glucose levels and insulin release (Liu et al., 2016). Moreover, low B6 levels have been associated with diabetic complications, such as neuropathy and retinopathy (Ellis et al., 1991; Nix et al., 2015), and to help in reducing diabetes complications (Kannan and Jain, 2004). Serotonin improves insulin sensitivity through serotonylation of Rab4, which likely represents the converging point between insulin and serotonin signaling cascades (Al-Zoairy et al., 2017). Bioinformatic and network pathway analysis carried out using IPA identified dysregulation of insulin as the central node in the pathway related to IR (Figure 8A). The second node with the highest connectivity in the network was serotonin, which showed that the highly interconnected regulation of serotonin with insulin altering the insulin signaling pathway. Moreover, 5-hydroxyindoleacetic acid (5HIAA), a breakdown product of serotonin, is down-regulated in the urine of diabetic patients. Serotonin plays a key role in controlling insulin secretion and its absence could lead to diabetes (Robinson, 2009). Elevation of the brain serotonin level may be regarded as an effective approach to treat T2DM and its complications (Derkach et al., 2015).

Branched-chain amino acids, including essential amino acids, play key roles in the energy homeostasis regulation, nutrition metabolism, gut health, immunity, and diseases. A positive association between increased circulating BCAAs with higher T2DM risk (Lotta et al., 2016) and IR in obese or diabetic patients (Zhao et al., 2016) has been reported. Similarly, AAA are strongly associated with the development of T2DM and IR (Yang et al., 2018). In a recent prospective study, aimed at identifying novel metabolic biomarkers predictive of future diabetes in 11,896 young adults from four Finnish cohorts, the strongest biomarkers of diabetes risk were BCAA and AAA (Ahola-Olli et al., 2019). In another targeted metabolomics platform, BCAA, AAA [phenylalanine (Phe) and tyrosine (Tyr)], Glu/Gln, Met, and C3 and C5 acylcarnitines were found to be strongly associated with IR (Newgard et al., 2009; Chen et al., 2016). A dramatic drop in BCAA and C3 and C5 acylcarnitines was observed in obese cases with T2DM following gastric bypass or gastric sleeve (Laferrere et al., 2011; Magkos et al., 2013). Furthermore, Leu, Ile, Val, Phe, and Tyr levels in plasma were also found to be associated with future development of T2DM (Wang et al., 2011; Chen et al., 2016). However, results seem to be controversial in different races, diets, and distinct tissues (Zhao et al., 2016). Recently, Lone et al. reported an association between five essential [Ile, Leu, lysine (Lys), Phe, and Val] and five non-essential [alanine (Ala), Glu, Gln, glycine (Gly), and Tyr] amino acids and the prevalence of T2DM (Lu et al., 2019). Association with the incidence of T2DM and four essential (Ile, Leu, Trp, and Val) and two non-essential (Gln and Tyr) amino acids was also reported while the accumulation of Gln and Gly was associated with T2DM lower risk (Lu et al., 2019). Moreover, abnormal circulating amino acid profiles in obesity, T2DM, and metabolic syndrome as measured by UPLC-TQ-MS demonstrated a decline in serum Gly and an increase in Val, Ile, Glu, and proline (Pro) in obesity, metabolic syndrome, and T2DM (Okekunle et al., 2017). In our study, arginine (Arg), Ser, Asp, and Gly were inhibited in T2DM whereas Asn, Gln, and His serum levels were lower in IR. The metabolomics profile of T2DM in our study showed the involvement of AAA through the presence of their intermediates. Metabolites of Phe, namely, homogentistic acid and 4-Amino-4-deoxychorismate, and of Tyr; 3,4 hydroxymandelic acid and hydroquinone, were identified. All these variations stem from using different methodologies and instrumentations and the fact that T2DM is a disease caused by a complex interchange between genetic, epigenetic, and environmental factors (diet and activity level), and that diabetes affects many major organs, including the heart, blood vessels, nerves, eyes, and kidneys. Additionally, genetic factors can make some people more vulnerable to diabetes. Thus, it is hard to identify distinct metabolic patterns that could serve as metabolic biomarkers for IR and T2DM. Moreover, in our study, we excluded age, BMI, and LDL-C in the analysis. This resulted in a reduction of the number of metabolites correlating with IR and T2DM. Age was considered as a confounder factor since both obese and T2DM groups were significantly older than the normal weight group. This represents a limitation for the study. Other confounders could impact IR and T2DM metabolome panels.

An interesting metabolite that was inhibited in T2DM in our study is pipecolate or 2 aminoadipic acid (2-AAA) which is an intermediate of the Lys degradation pathway. Previous studies have shown that circulating pipecolate levels were strongly associated with obesity and metabolic syndrome and had the ability to predict the risk of future T2DM, HG, increasing insulin secretion in early IR, and had a lesser role in the setting of advanced IR or T2DM (Wang et al., 2013; Libert et al., 2018). In turn, pipecolate reported to enhance insulin secretion in cell-based, islet, and animal model systems (Wang et al., 2013), and contribute to a compensatory mechanism by up-regulating insulin secretion to maintain glucose homeostasis in early IR. It has been found to independently act on β-cells of the pancreas to regulate the release of insulin at glucose-dependant concentrations. It augments the release of insulin at 2.5 mmol/L whereas higher levels of glucose (>11.1 mmol/L) inhibit this augmentation. This was also seen in our metabolic profile where levels of pipecolic acid were reduced in T2DM compared to the obese while those in the obese were higher than their lean counterparts. In another study, Wang et al. showed that treatment of diet-induced obesity with pipecolic acid significantly reduced body weight, fat accumulation, and lowered fasting glucose. Pipecolate regulating glycolipid metabolism is independent of diet and exercise, implying that improving its level can be a mean to treat diabetes (Xu et al., 2019). Our observations of a decrease in the levels of pipecolate in established T2DM cases are consistent with the previous findings and a possible explanation could be that pipecolate contribution to maintaining glucose homeostasis is overcome in established diabetes. In this scenario, an early measurement of pipecolate could serve as a novel potential metabolic marker of hyperglycemia that would predict a predisposition to T2DM and would be used in diabetes risk assessment.

Few dipeptides are known to be associated with physiological or cell-signaling effects such as ophthalmic acid in cystic fibrosis (DiBattista et al., 2019). However, most are simply short-lived intermediates on their way to degradation pathways following further proteolysis. His-Ala is a dipeptide resulting from incomplete breakdown of protein digestion or protein catabolism. In this study, His-Ala is inhibited in T2DM. Interestingly, His-Ala has been patented for reducing uric acid (Patent# JP2004359663A). High levels of uric acid in the blood are associated with increased risk of developing diabetes (Xiong et al., 2019). On the other hand, T2DM is associated with high serum uric acid levels and thus levels of His-Ala could be inhibited by uric acids. Peptidases play a pivotal role in the production, degradation, and regulation of peptides *in vivo* (Tiruppathi et al., 1990; Rosenblum and Kozarich, 2003). Prolyl peptidases are characterized by a biochemical preference for cleaving Pro-containing peptides. Prolyl peptidases family includes prolyl endopeptidase, prolyl endopeptidase-like, dipeptidyl peptidase 4 (DPP4), DPP7, DPP8, DPP9, and fibroblast activation protein (Lone et al., 2010). DPP4 (also known as CD26) selectively cleaves dipeptides from peptides and proteins containing Pro or Ala in the N-terminal penultimate position (Gorrell et al., 2001; Kirby et al., 2009). This proteolysis can alter activities of target substrates, including the functional activity of bioactive peptides or facilitated degradation of macromolecules by other peptidases. DPP4 plays a major role in glucose and insulin metabolism (Rohrborn et al., 2015). DPP4 does cleave Ala containing dipeptides and thus lowering levels of His-Ala could result from higher levels of DPP4 in diabetes. Similarly, six serum dipeptides had concentrations lower in T2DM than obese and control groups. These include Glu-Ser, Gly-Ala, Prol-Gly, Leu-Gln, Arg-Pro, and Arg-Glu dipeptides. Lower serum levels of these peptides could result from increased peptidases and could represent a target for T2DM treatment. On the other hand, other serum dipeptides concentrations are increased in T2DM. These include dipeptides Cys-Gly, Ala-Ser, Ala-Glu, and BCAA-Pro dipeptides, Pro-Val, Pro-Leu, Pro-Ile. Among these dipeptides, only Ala-Glu and Ala-Ser are glucose-dependent metabolites. Further investigations are needed to clarify the role of these dipeptides in diabetes.

We identified phosphoethanolamine (PE) and diethanolamine in T2DM metabolomics profile. PE and diethanolamine are substrates for the synthesis of phospholipids, phosphatidylcholine (PC), and phosphatidylethanolamine (PtdE). PE is the crucial metabolite determining the rate-limiting step of the reaction, to produce CDP–ethanolamine via the cytidine dependant CDP-ethanolamine metabolic pathways, which, together with DAG, generates PtdE (Steenbergen et al., 2005). Phospholipids are major components of all cellular membranes with PtdE being the major phospholipid of the mitochondrion. The CDP-ethanolamine pathway is an important regulator of hepatic lipid homeostasis (Leonardi et al., 2009). The involvement of PtdE and more importantly the PtdE/PC ratio points to alterations in phospholipid pathway that regulates muscle IR, insulin sensitivity, and to the presence of endoplasmic reticulum stress and mitochondrial dysfunction (Fu et al., 2011; Meikle et al., 2013). Interestingly, we also identified two metabolites participating in the folate metabolism, namely, 7-craboxy-7-carbaguanidine and pyrimidodiazepine. Folate is a cofactor known to regulate major metabolic pathways including the phospholipid pathways. Decreased levels of folate in an animal study showed optimal folate levels determine the synthesis of PC via the methylation of PtdE, giving further credence to our findings of dysregulation of phospholipids with T2DM (Zhao et al., 2018). Besides, folate deficiency is known to predispose to obesity, lipid disorders, and T2DM, and an increase in the metabolites involved in its synthesis may account for a compensatory mechanism (Li et al., 2017).

Another interesting finding in our metabolomic profiling of T2DM is the dysregulation of the pyrimidine metabolic pathway; cytidine, 5-amino-6-(5’-phospho-D-ribitylamino) uracil, uracil, and aminoacrylate. Cytidine is an important molecule required for the synthesis of di/triphosphates that act as a fundamental source of energy for cellular reactions and are involved in signaling pathways. Aside from being intracellular energy molecules, nucleotides play an important role as extracellular signaling molecules and have been described for adenosine (Burnstock and Novak, 2013) and uridine (Yamamoto et al., 2010) in terms of glucose regulation, IR, and diabetes. We found a decrease in the levels of uracil in our study. Previous studies have shown changes in the levels of nitrogen compounds, such as nucleotides, nucleosides, and their metabolites, vary considerably, depending on the degree of IR in obese subjects (Yang et al., 2018). By the same inference, this role can be extended to cytidine which is being reported in this study. Network pathway analysis relating to HG centered around dysregulation of signaling pathways related to ERK, p38 MAPK, and Akt (Figure 8B). Ser and threonine kinases are known regulators of cellular functions that include glucose metabolism, glycogen synthesis, protein synthesis, cell proliferation, cell hypertrophy, and cell death. These signaling pathways also regulate proinsulin, another node identified in the network map. Interactions of Akt and p38 MAPK are important mediators of insulin action via modulating INSR substrate and GLUT 4 activity. HG alters the Akt and members of the MAP kinase family signaling proteins there contributing to type 2 diabetes (Rane et al., 2001). Interpretation of the metabolome database thus contributes to the development of a comprehensive and accessible dataset of detected metabolites in obese and obese T2DM plasma samples for the discovery of disease associations and diagnoses in future research.

The current study is the lack of an independent external validation cohort. We plan to expand this work in the future by recruiting more subjects from multiple centers. Another limitation of the current study is the coverage of small molecules. While CIL LC-MS offers a high-coverage analysis of a chemical-group-based submetabolome, the current work only profiled the amine/phenol submetabolome. Other submetabolomes (e.g., acids, carbonyls, and hydroxyls) need to be examined in the future. In addition, we did not examine the lipidome. The results reported in this study demonstrated significant changes in comparative groups, suggesting that global metabolome and lipidome analysis is warranted in future studies where larger cohorts of samples will be collected in order to increase both coverage and statistical power.

## Conclusion

Metabolomics is an emerging approach for studying metabolic changes connected to disease development and progression. Metabolite-profiling techniques improvement is providing the increased extent of coverage of the human metabolome and advances have led to the application to defining predictive biomarkers and pathways for diseases including T2DM. Different metabolomic profiles have been reported in obesity and T2DM. This study identified nine metabolomics profile for IR and 42 for T2DM after the exclusion of confounders (age, BMI, and LDL-C). Identification and characterization of the metabolomic pattern in obese subjects might aid in identifying subjects at a high risk of developing metabolic diseases such as T2DM, thus allowing early treatment intervention. Future studies are required to establish causal relationships between metabolic biomarkers identified in this study for IR and T2DM and to examine their predictive values for developing T2DM.

## Data Availability Statement

The datasets presented in this study can be found in online repositories. The names of the repository/repositories and accession number(s) can be found in the article/Supplementary Material.

## Ethics Statement

The studies involving human participants were reviewed and approved by King Abdulaziz Medical City Ethics Committee (Protocol # RC12/105), and King Faisal Specialist Hospital and Research Center (KFSHRC) (RAC# 2170 013). The patients/participants provided their written informed consent to participate in this study.

## Author Contributions

ASA and AAR designed the projected, performed the data analysis, supervised the clinical and experimental activity, drafted the manuscript, and approved the final version. XG and LL generated the metabolomics data and performed part of the metabolome data analysis. AM and HB interpreted the biochemical pathways and helped in the final draft. MA, AwA, and MZ recruited the patients and collected the clinical data. All the authors approved the final version.

## Conflict of Interest

The authors declare that the research was conducted in the absence of any commercial or financial relationships that could be construed as a potential conflict of interest.

## Footnotes

1 www.metaboanalyst.ca

## Abbreviations

AAA: aromatic amino acids
ACN: acetonitrile
Asn: asparagine
BCAA: branched-chain amino acid
BMI: body mass index
CIL: chemical isotope labeling
DnsCl: dansyl chloride
FA: formic acid
Gln: glutamine
HG: hyperglycemia
His: histidine
HOMA-IR: homeostatic model assessment
INSR: insulin receptor
IR: insulin resistance
IRS-1: insulin receptor substrate-1
LC-MS: liquid chromatography-mass spectrometry
LDL: low-density lipoprotein
LHD: lower high-density lipoprotein
NEFAs: non-esterified fatty acids
PLS-DA: partial least squares discriminant analysis
T2DM: type 2 diabetes mellitus
Trig: triglycerides.
