# Supplementary material for: Distinctive Metabolomics Patterns Associated With Insulin Resistance and Type 2 Diabetes Mellitus
Source: Front Mol Biosci. 2020 Dec 14;7:609806. doi: 10.3389/fmolb.2020.609806 (PMC7768025; doi:10.3389/fmolb.2020.609806)
Supplement: Supplementary file 5 [file Presentation_1.pdf]

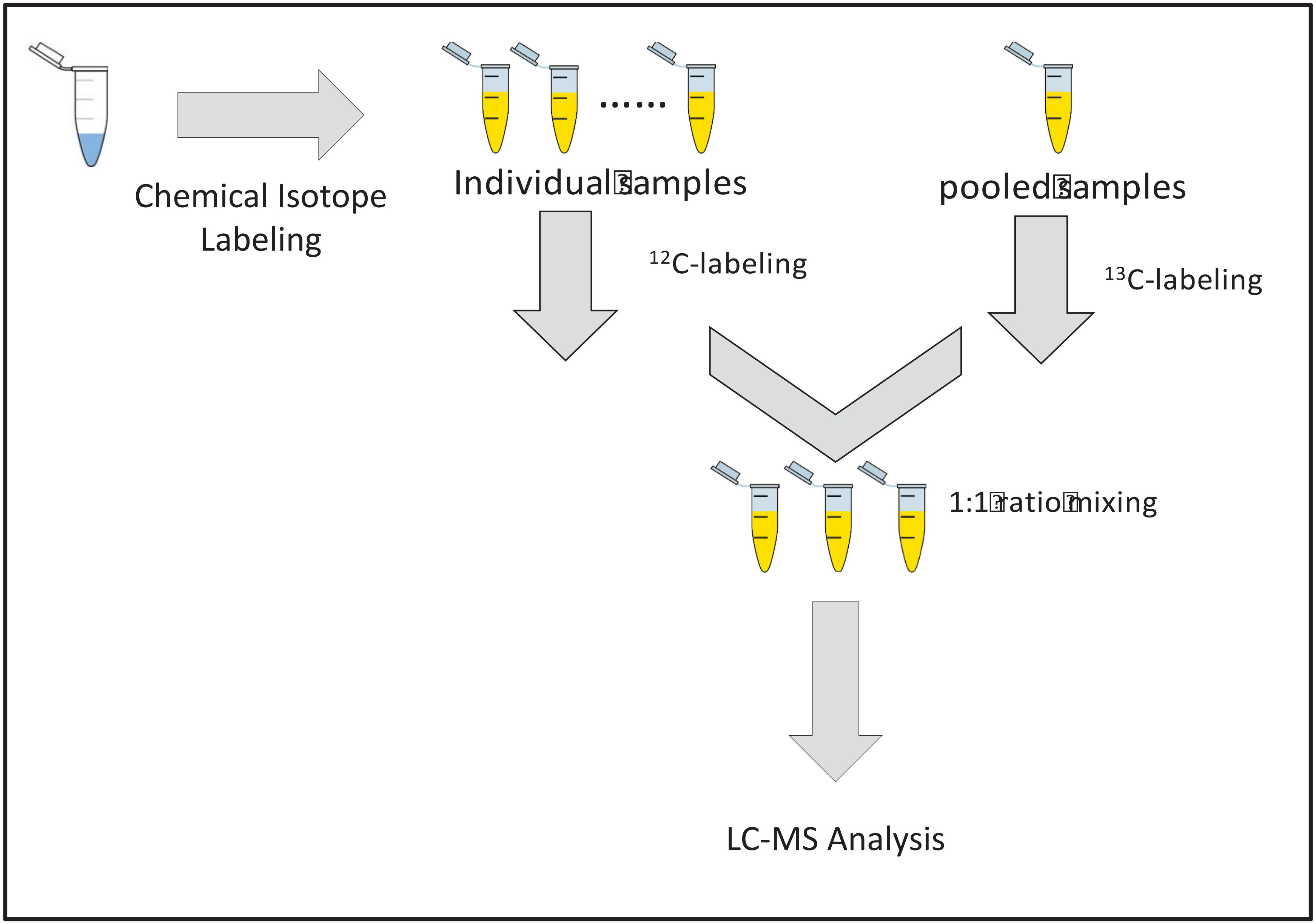

Figure S1: Schematic diagram of overall workflow of chemical isotope labeling.

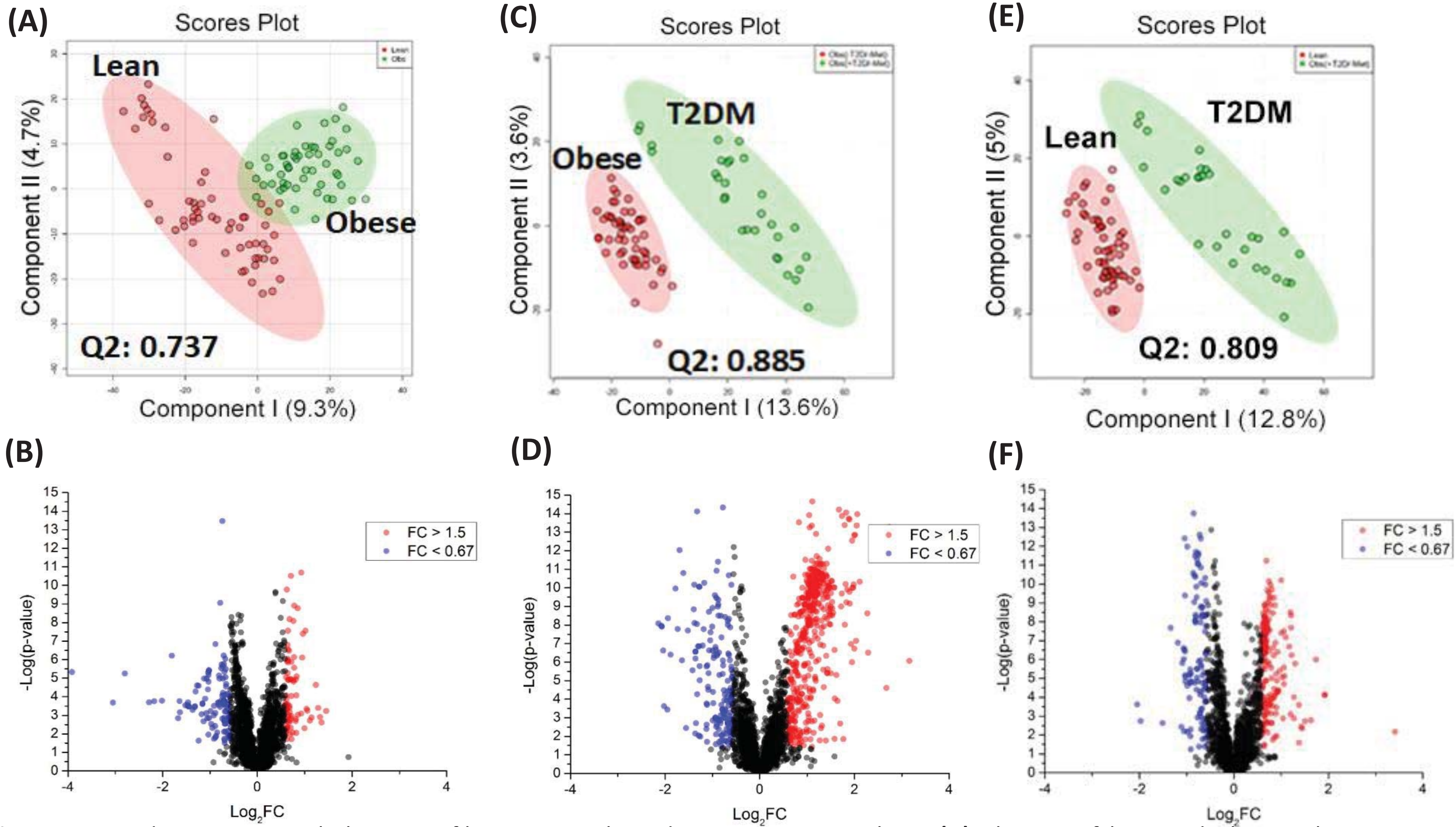

Figure S2: Study groups metabolomic profiles were evaluated using PLS-DA analysis. **(A)** Clusters of lean and obese subjects were separated (Q2=0.737, R2=0.972). **(B)** Volcano plot of obese versus lean group. 78 metabolites were up- and 111 were down-regulated in obese group with fold change and FDR adjusted p-value at the cut-off 1.5 (or 0.67) and 0.05, respectively. **(C)** Clusters of Obese and T2DM groups were separated (Q2=0.885, R2=0.985). **(D)** Volcano plot of T2DM versus obese groups. 459 metabolites were up- and 166 were down-regulated in T2DM group with fold change and FDR adjusted p-value at the cut-off 1.5 (or 0.67), and 0.038, respectively. **(E)** Clusters of lean and T2DM groups were separated (Q2=0.809, R2=0.977). **(F)** Volcano plot of lean versus T2DM groups. 189 metabolites were up- and 117 were down-regulated in T2DM group with fold change and FDR adjusted p-value at the cut-off 1.5 (or 0.67), and 0.068, respectively.

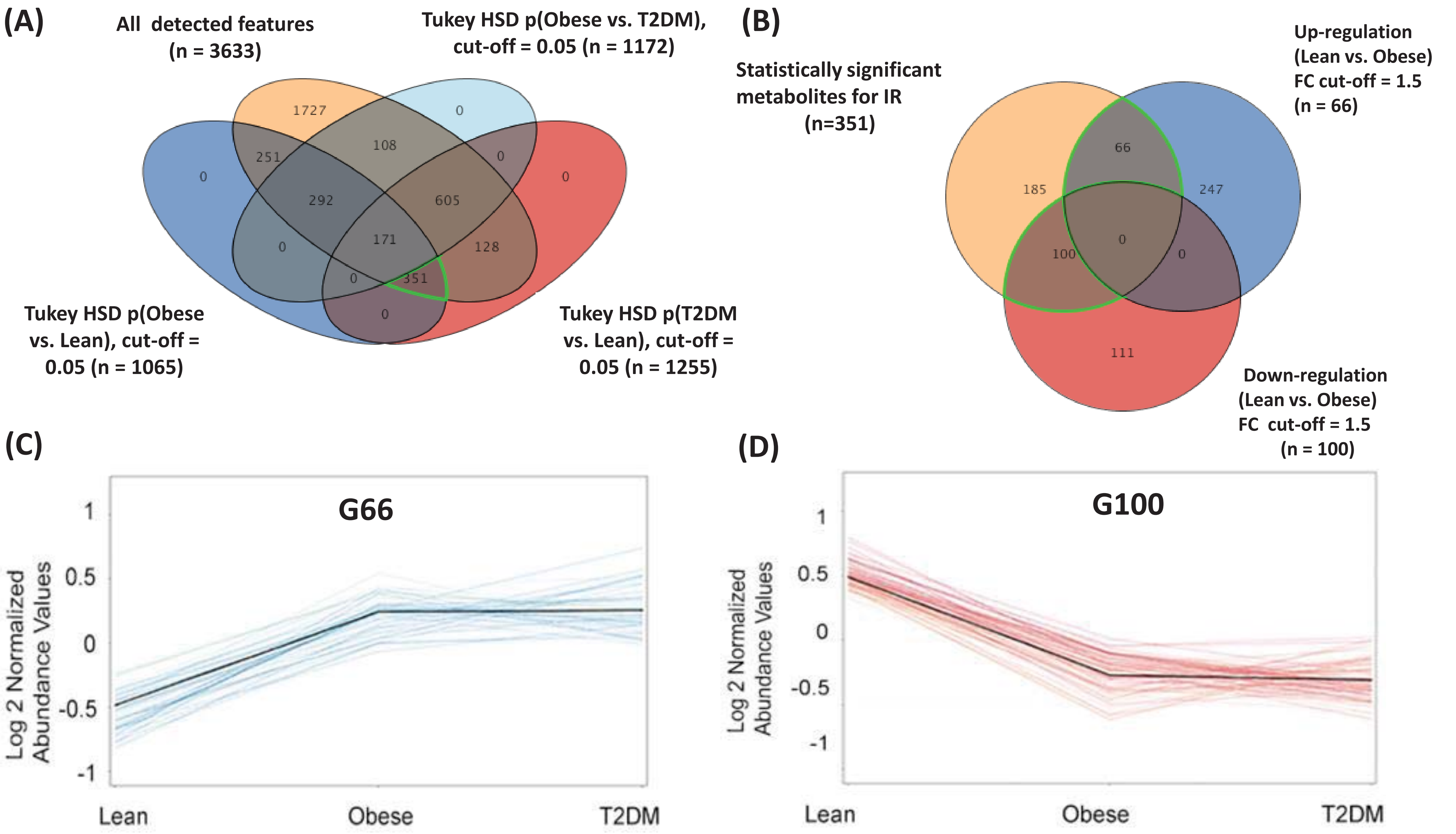

Figure S3: IR model based on both identified and unidentified features detected by the metabolomics platform. (A) Among the 3633 detected features, only 351 (G351) metabolites had an IR profile (statistical significance between lean and obese, and lean and T2D, and insignificance between obese and T2D (FDR-Corrected p-value <0.05). **(B)** Fold change analysis separates the group of 351 significant metabolites into up- and down-regulated features in lean compared to obese and T2DM (66 and 100 metabolites, respectively) with 1.5 as fold change cutoff. **(C)** Representative profile of the up-regulated (G66) and **(D)** down regulated metabolites (G100).

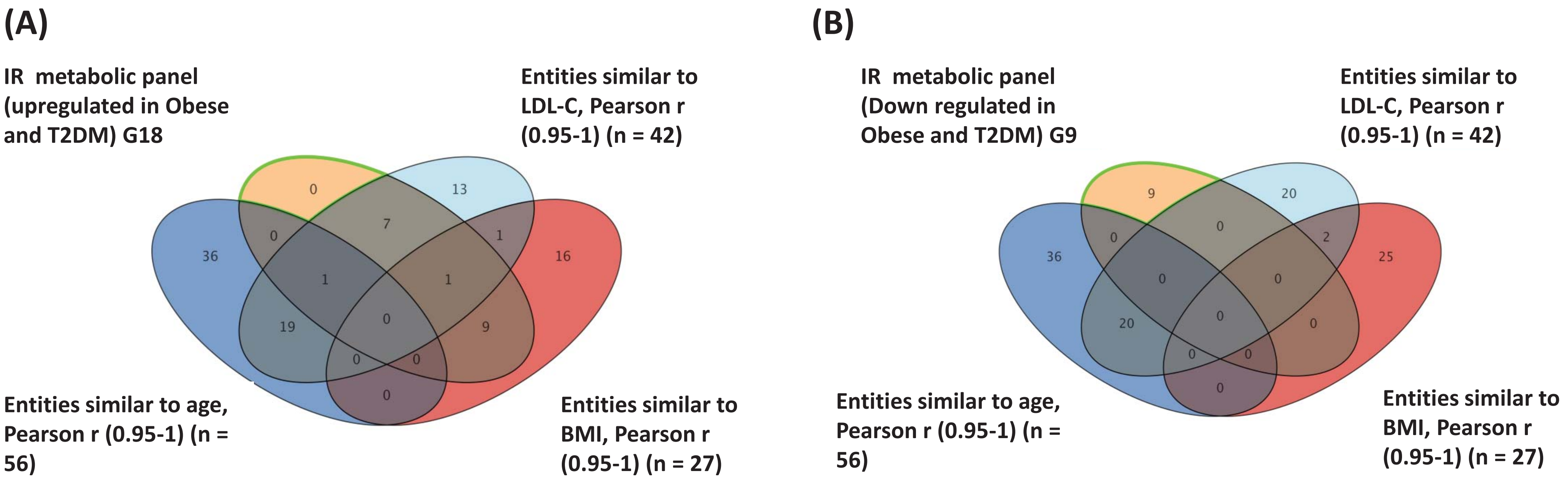

Figure S4: BMI-, LDL-C-, and age- independent metabolic pattern for **(A)** IR upregulated metabolic panel (G18), and **(B)** IR down regulated metabolic panel (G9).

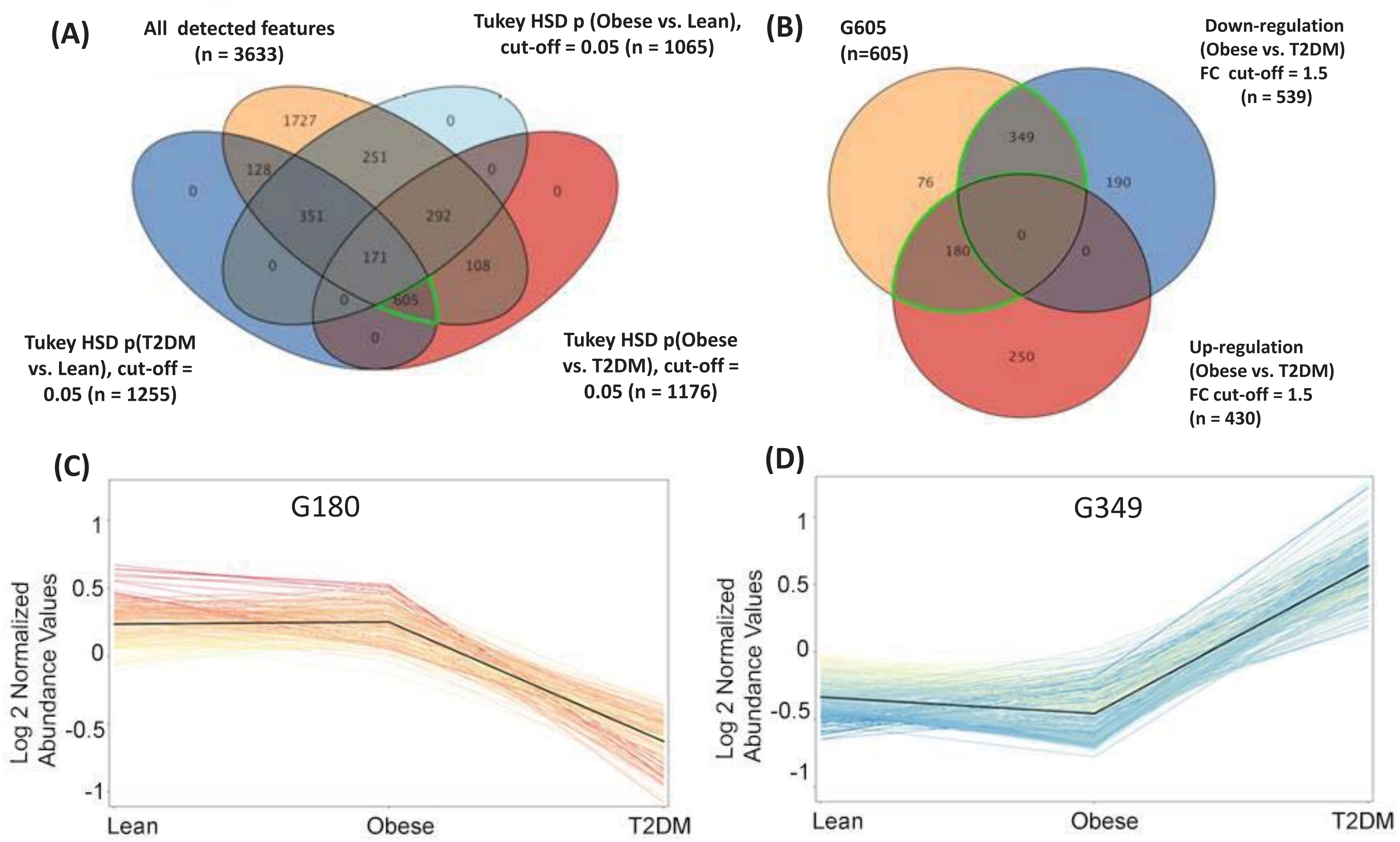

Figure S5: T2DM metabolic model based on both identified and unidentified features detected by this metabolomics platform. (A) Among the 3633 detected features, only 605 metabolites were considered a pattern for T2DM. Statistical significance between lean and T2DM, and obese and T2DM, and insignificance between lean and obese (FDR-Corrected p-value <0.05)); **(B)** Fold change analysis with cutoff 1.5 separates these significant metabolites into up- and down-regulated groups when T2DM compared to obese (349 and 180 metabolites, respectively); **(C)** Representative profile of the down-regulated metabolites (G180) and **(D)** up-regulated metabolites (G349).

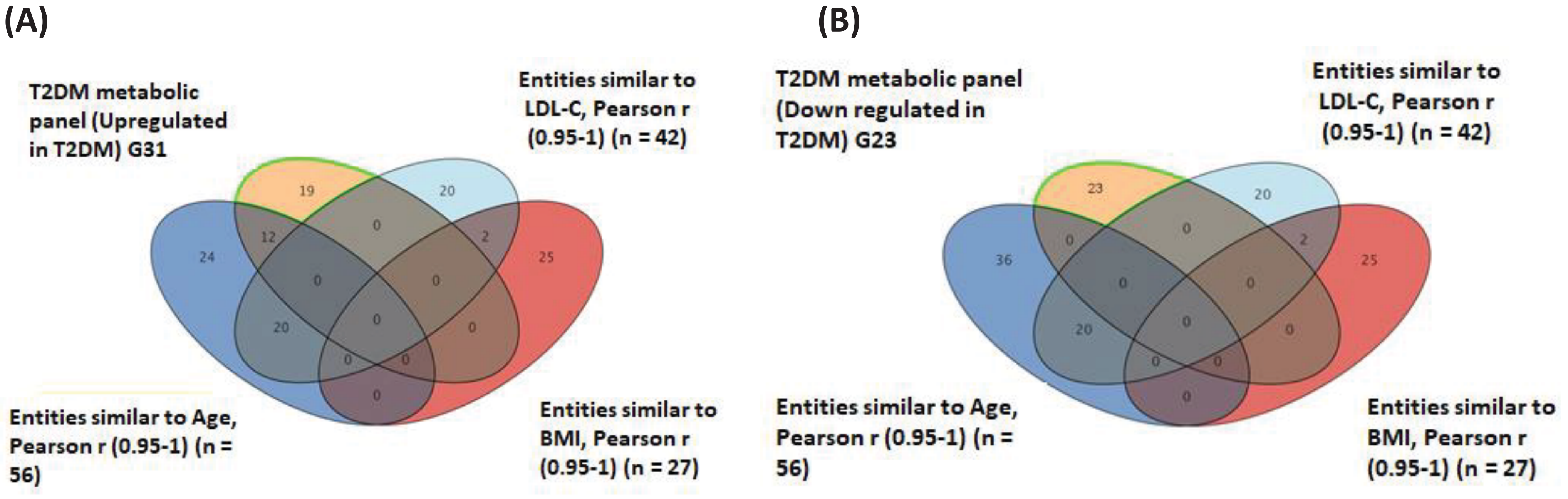

Figure S6: BMI-, LDL-C-, and age- independent metabolic pattern for (A) T2DM upregulated metabolic panel (G31), and (B) T2DM down regulated metabolic panel (G23).

(A)

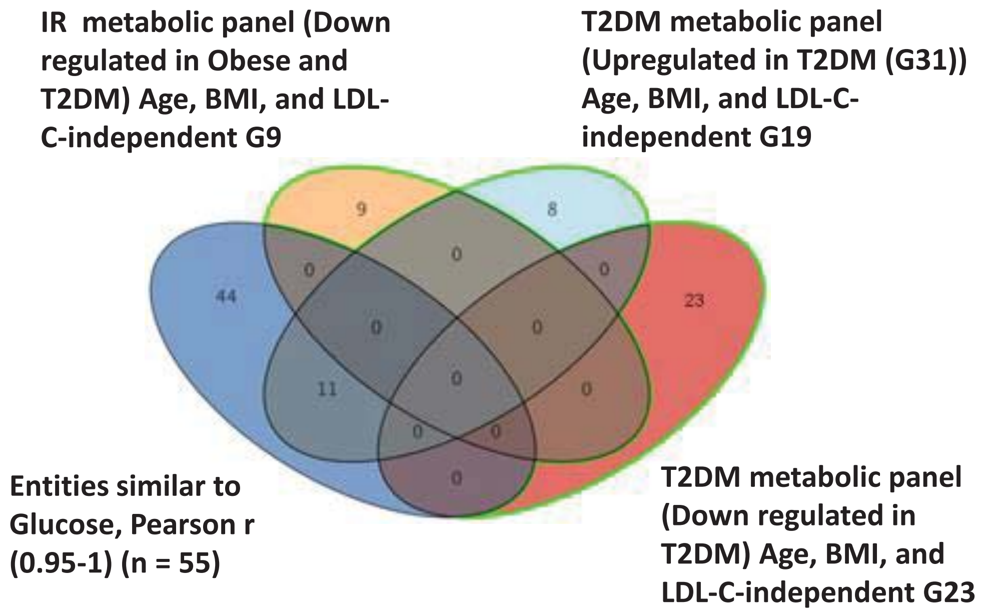

(B)

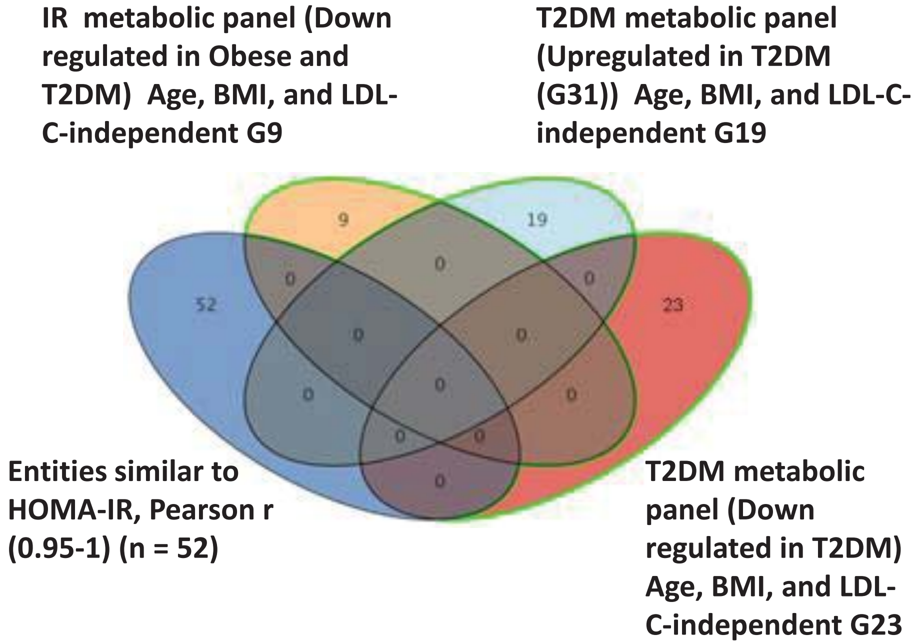

(C)

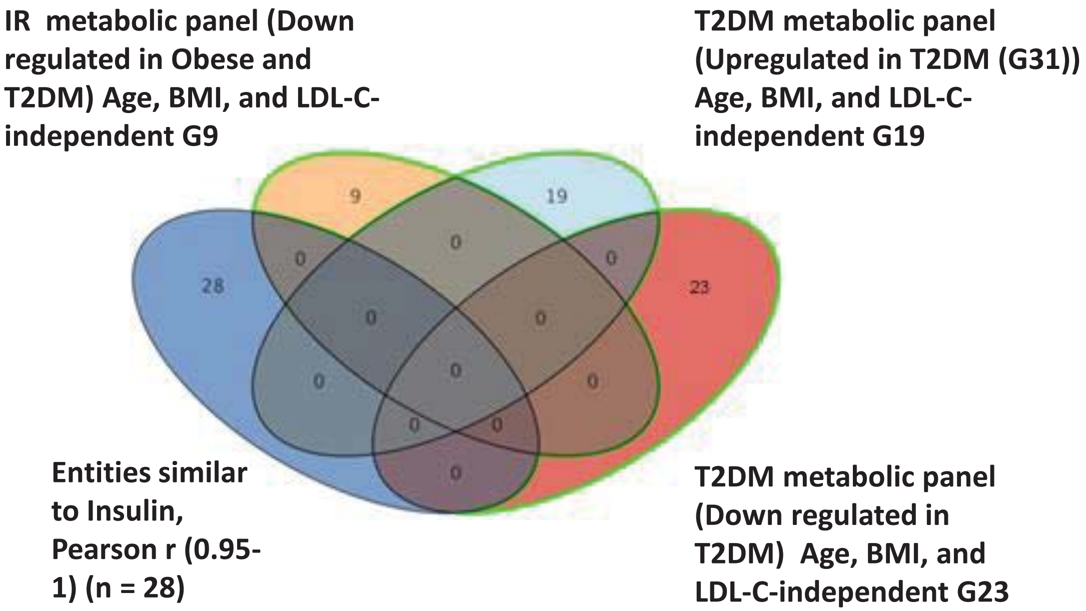

Figure S7: IR and T2DM metabolic panels, after excluding age, BMI, and LDL-C related metabolites, correlated to (A) Glucose, (B) HOMA-IR, (C) Insulin.

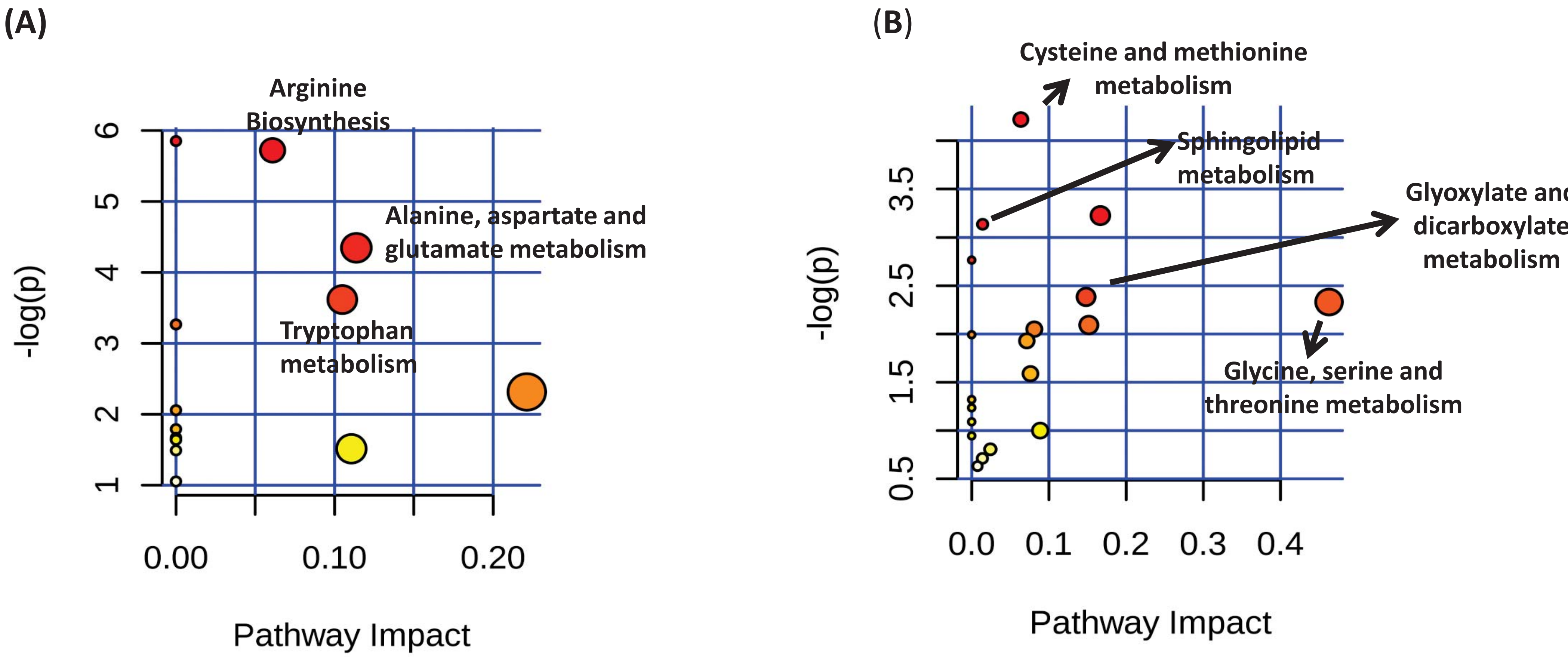

Figure S8: Pathway impact plots that display the most important pathways involved in the metabolic changes due to **(A)** IR and **(B)** T2DM mechanisms.

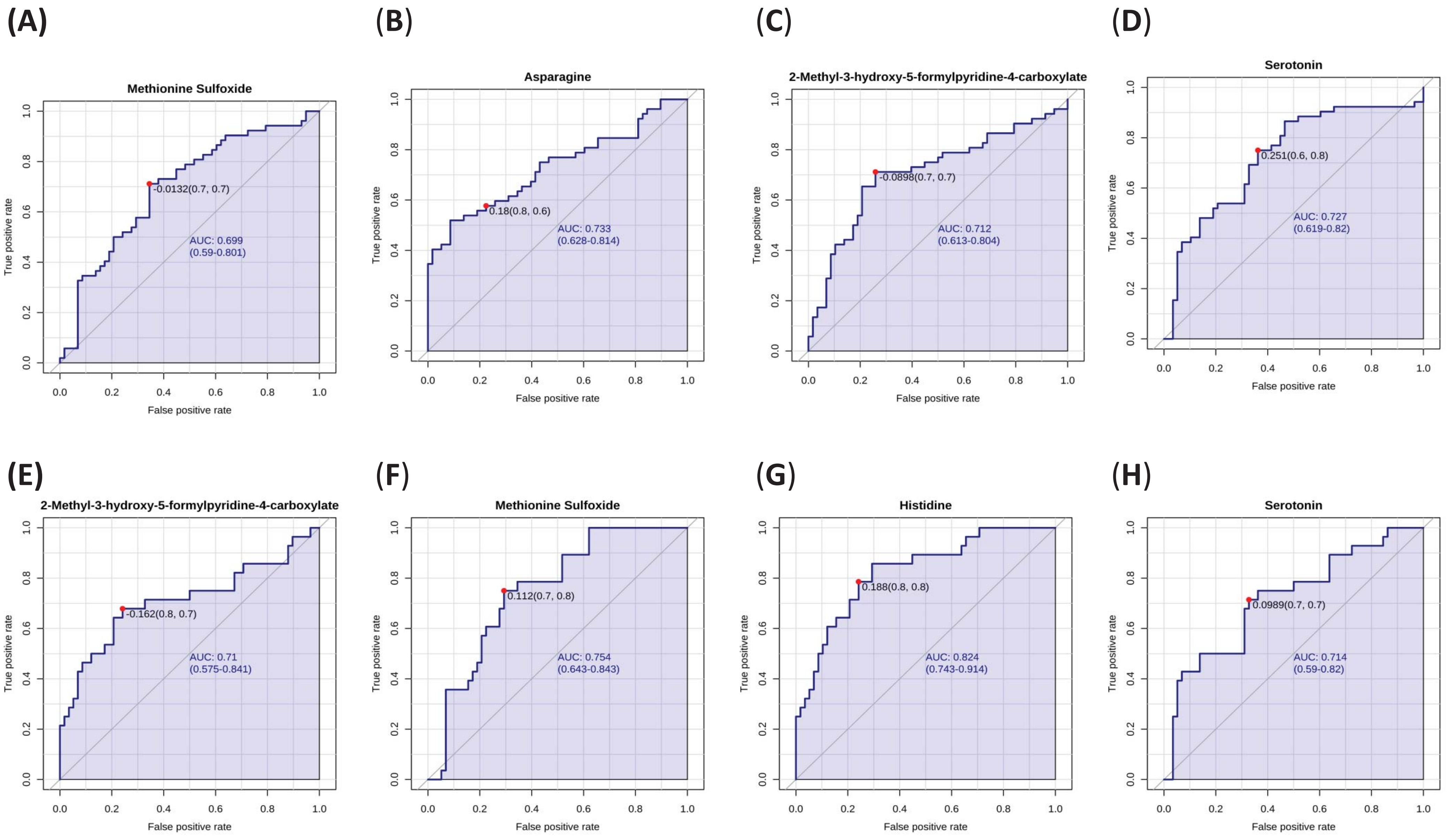

Figure S9: Representative individual ROC curves for IR potential metabolic biomarkers predicted in lean compared to obese group **(A)** Methionine sulfoxide, **(B)** Asparagine, **(C)** 2-methyl-3-hydroxy-5-formylpyridine-4-carboxylate, and **(D)** Serotonin, and in Lean compared to T2DM **(E)** 2-methyl-3-hydroxy-5-formylpyridine-4-carboxylate, **(F)** Methionine sulfoxide, **(G)** Histidine, and **(H)** Serotonin.

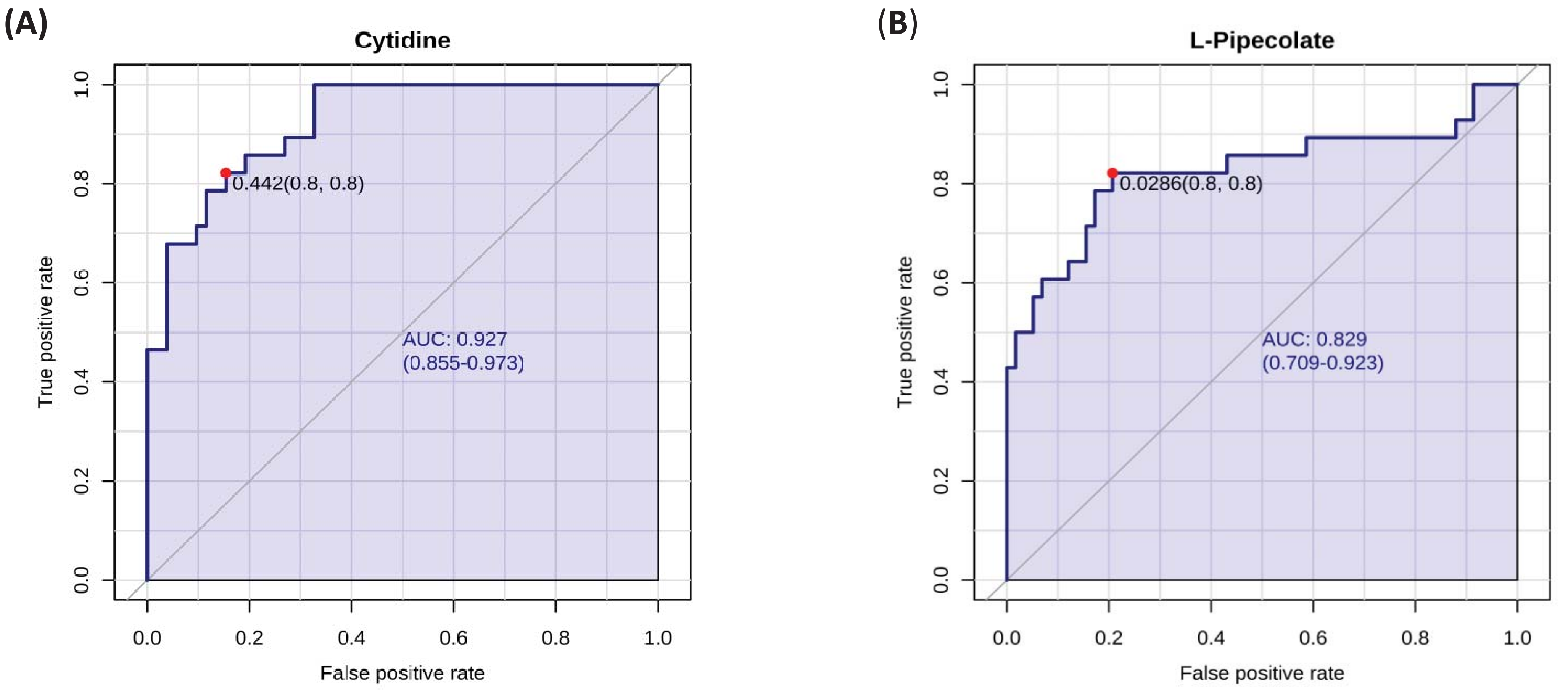

Figure S10: : Representative individual ROC curves for T2DM potential metabolic biomarkers predicted in lean compared to T2DM and Obese compared to T2DM (A) Cytidine, and (B) Pipecolate.
